# Supplementary material for: WalK(S221P) Mutation Promotes the Production of Staphylococcus aureus Capsules Through an MgrA-Dependent Pathway
Source: Microorganisms. 2025 Feb 25;13(3):502. doi: 10.3390/microorganisms13030502 (PMC11944577; doi:10.3390/microorganisms13030502)
Supplement: Supplementary file 1 [file microorganisms-13-00502-s001.zip › microorganisms-3477409-supplementary.pdf]

Supplementary Materials

Supplementary Figures

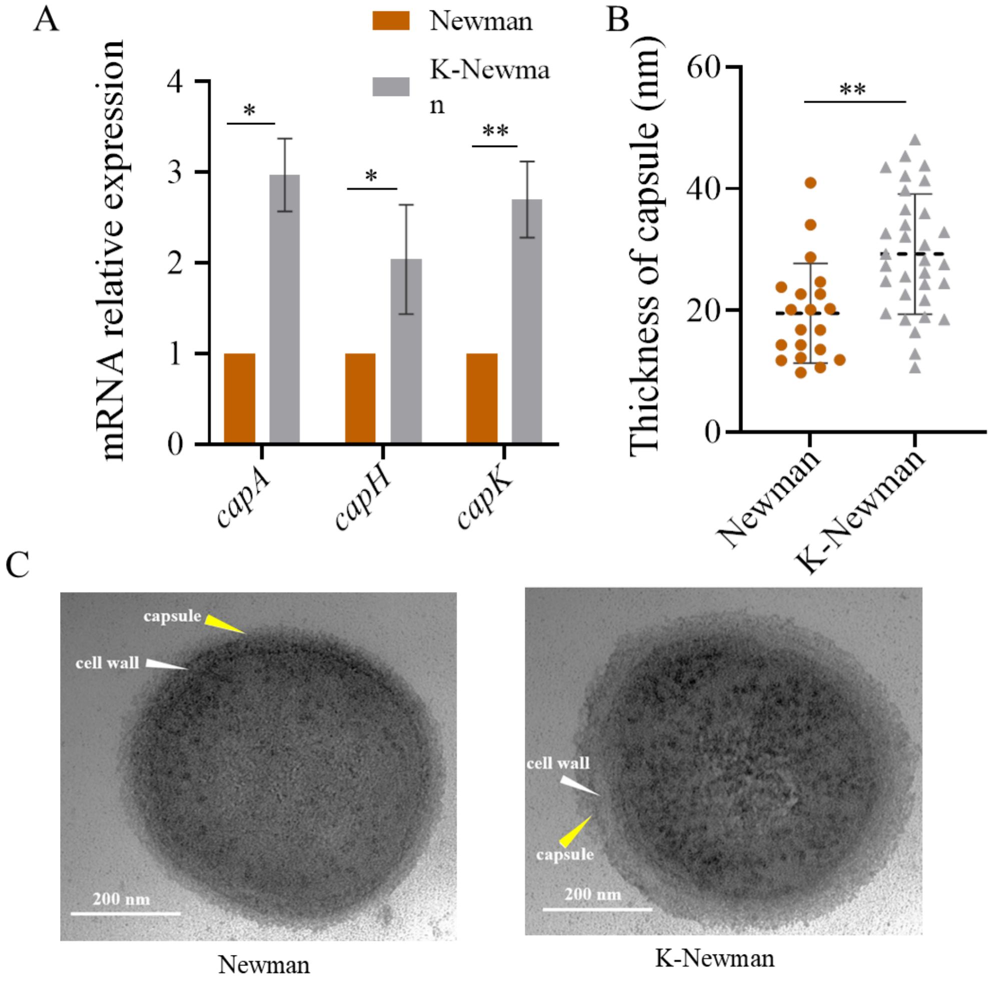

Figure S1. WalK(S221P) mutation promotes capsule production of *S. aureus* strain Newman. (A) RT-qPCR detection of *cap* genes *capA, capG,* and *capL* in Newman and K-Newman. The *gyrB* was used as a reference gene for normalization. The data are expressed as the mean ± SD (n = 3). (B) Capsule thickness comparison. Data were expressed as mean ± SD (Newman, n = 20; K-Newman, n = 32). (C) Representative TEM images of Newman and K-Newman. The cell wall and capsule are indicated by white and yellow triangles, respectively. Statistical significance in A and B was calculated by Student's t-test, * represents $P < 0.05$, and ** indicates $P < 0.01$.

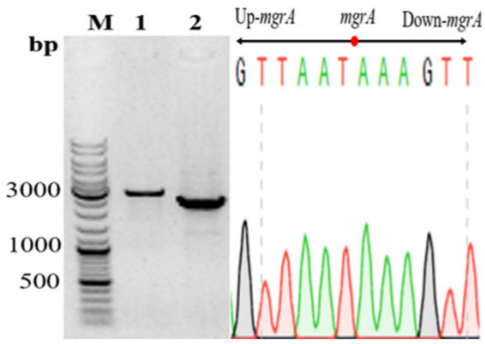

Figure S2. Characterization of the *mgrA* deletion mutant XN108Δ*mgrA*. PCR showing a shorter product derived from XN108Δ*mgrA* (lane 2) than that of the wild-type XN108 (lane 1, Left panel). Diagram showing the sequencing analysis of the *mgrA* gene deletion in *S. aureus* XN108Δ*mgrA* (Right panel).

Table S1. Primers used in this study.

Prime	Oligonucleotide (5′-3′)	Length
pET28a-*mgrA*-F	cagcaaatgggtcgcggatccatgtctgatcaacataatttaaaagaac	444 bp
pET28a-*mgrA*-R	tgcggccgcaagcttgtcgacttatttttcctttgtttcatcaaatgc	
cap-F-biotin	atcatctaactcacctgaaattacaaaagt	348 bp
cap-F	atgccacataaacttaaagtcgataatc	348 bp
cap-R	ttctaatgtactttccattatttacctccc	
hu-F	aaaaagaagctggttcagcagtag	125 bp
hu-R	tttacgtgcagcacgttcac	
*ag*r-F-biotin	ttattttttagtgaatttgttcactgtgtcg	222 bp
agr-R	aacgactagttaagaaaaattggaaaataaatg	
mgrA-F-biotin	tttatattctcttttctattacagttatttttaaa	227 bp
mgrA-F	tttatattctcttttctattacagttatttttaaa	227 bp
mgrA-R	taaagttctcctccagacatactatcc	
pOS-*cap*-F	ccggaattcatgccacataaacttaaagtcgataatc	348 bp
pOS-*cap*-R	gcgggatccggttctaatgtactttccattatttacctccc	
pOS-*mgrA*-F	ccggaattcggttttaccactacccaaaaatccg	333 bp
pOS-*mgrA*-R	gcgggatccggattatgttgatcagacattaaagttctcctc	
RT-*capA*-F	aattattagcgctattgttacatttttcg	200 bp
RT-*capA*-R	catttaagtcctttgacacctcatct	
RT-*capH*-F	ttggtaatgtcagatttaatgagataaatcaaaaat	196 bp
RT-*capH*-R	tcaatttcatttaaatatttcggtgtttcttcc	
RT-*capK*-F	aatcatcactaccccaattattacaagac	166 bp
RT-*capK*-R	ttgtgaaagtatattagcactacggtcatt	
RT-*mgrA*-F	ttaacttcatcttgcgataaagaagaag	151 bp
RT-*mgrA*-R	aagtagacttaattaagcgtgaacgt	
RT-*saeR*-F	cgccttaactttaggtgcagatgac	196 bp
RT-*saeR*-R	acgcatagggacttcgtgaccatt	
RT-*saeS*-F	cacctttagcaagcattatttcatattctgaag	201 bp
RT-*saeS*-R	gtaattggtctagttgtattaattctggggg	
RT-*rot*-F	tgggagatgtttagcatgaaaaaagta	174 bp
RT-*rot*-R	cgtcatagaacctttttgccataaagtta	
RT-*spoVG*-F	cgtaattcatgatttacgtgtaattgaagga	206 bp
RT-*spoVG*-R	ctgaatcttctgatgtagcgtttttatct	
RT-*rbsR*-F	gatggcgaatatgtcgactcg	213 bp
RT-*rbsR*-R	ctgatgccatgttcaattagtcctc	
RT-*codY*-F	gtatttattgtatcgcgtcgaggtaa	206 bp
RT-*codY*-R	atgaataattctctgttttcaggtggg	
RT-*gyrB*-F	aaatcgcctgcgttctagag	121 bp
RT-*gyrB*-R	ccaggtaaattagccgattgc	
pBT2-*mgrA*-left-F	agctcggtacccggggatccattcaaattaatgcaggccaac	1026 bp
mgrA-left-R	atgtctggaggagaactttattaacttttgtcatgacaattaaag	
mgrA-right-F	taaagttctcctccagacatactatccg	1000 bp
pBT2-*mgrA*-right-R	ttgcatgcctgcaggtcgacatctctaaatgacacataacc	

pBT2-F	gagagtcattaccccaggcgtt	501bp
pBT2-R	gagcaactggatcgatccacag	
mgrA-left-out-F	atgcagctaatgaacaggc	2946 bp
mgrA-right-out-R	actccatccctcaatgtcac	
mgrA-in-F	gcgactttgtcagatgcattac	208 bp
mgrA-in-R	gggatgaatctcctgtaaacg	
pLI/*mgrA*-F	ccctttcgtcttcaagaattcggttttaccactacccaaaaatccg	739 bp
pLI/*mgrA*-R	tctagaggatccccgggtaccttatttttcctttgtttcatcaaatgc	
